# Diagnostic value of genetic testing in chorea: a retrospective monocentric study

**DOI:** 10.1007/s00415-026-13938-3

**Published:** 2026-06-19

**Authors:** Tekla Anna Fodor, Ivan Milenkovic, Alexander Zimprich, Christof Brücke

**Affiliations:** Department of Neurology, Medical University of Vienna, 1090 Vienna, Austria

**Keywords:** Chorea, Huntington’s disease, Movement disorders, Whole genome sequencing, Neurogenetics, Genetic testing

## Abstract

**Background:**

Chorea is a hyperkinetic movement disorder with a broad differential diagnosis, ranging from acute symptomatic causes to slowly progressive neurogenetic diseases. While Huntington’s disease (HD) remains the most prevalent hereditary form, numerous other genetic disorders may mimic its clinical presentation. A major diagnostic challenge arises in patients with a seemingly negative family history, which can obscure the suspicion of a genetic etiology. In patients with sporadic chorea, the potential contribution of genetic testing to the diagnostic process has not yet been systematically analyzed.

**Methods:**

We conducted a retrospective analysis of 81 patients presenting with chorea as a prominent symptom at the movement disorders outpatient clinic between 2013 and 2024. Clinical data, family history, laboratory results, imaging, and genetic analyses were evaluated. Genetic testing included a chorea-related gene panel and, if unremarkable, whole-exome or whole-genome sequencing.

**Results:**

Out of 81 patients, 44 presented with slowly progressive chorea and unremarkable family history of HD or chorea-related syndromes. After exclusion of secondary etiologies (n = 8), 36 patients remained, of whom 30 (83, 33%) received a confirmed genetic diagnosis. HD was the most frequent diagnosis (n = 20), followed by rare genetic disorders such as Spinocerebellar Ataxia Type 17 (n = 2), Wilson’s Disease (n = 2), Ataxia with Oculomotor Apraxia Type 2 (n = 1), C9orf72-related Neurodegeneration (n = 1), Choreoacanthocytosis (n = 1), KMT2B-related Dystonia (n = 1), ERCC4-related Neurodegeneration (n = 1), and Glutaric Acidemia Type 1 (n = 1).

**Discussion:**

These findings support the systematic use of genetic testing—even in apparently sporadic cases—and suggest that the prevalence of hereditary choreatic disorders, may be significantly underestimated.

## Background

Chorea is a hyperkinetic movement disorder with the characteristic features of involuntary, and unpredictable jerky movements that flow from one body part to another [1]. The presumed underlying pathophysiology is a dysfunction in the striatum of the basal ganglia, where structural damage or altered neurotransmitter function can lead to a reduced inhibitory output and excessive, uncontrolled motor activity [2]. As most of movement disorders, chorea can be an acquired syndrome or a result of genetic mutations [3, 4].

Huntington’s Disease (HD) is the most common and probably best known hereditary choreatic movement disorder with a prevalence of around 6–7 per 100.000 in Europe [5]. It is caused by a CAG trinucleotide repeat expansion in the *HTT* gene and presents with progressive generalized chorea, cognitive impairment, and psychiatric symptoms [6]. Recent advances in neurogenetics have identified a growing number of genetic disorders that clinically resemble HD. These HD phenocopies present a diagnostic challenge due to overlapping clinical features, de novo mutations, and the wide range of underlying etiologies [3, 7–9]. Although the exact prevalence of genetic choreatic disorders is unknown, HD phenocopies are estimated to account for 1–12% of patients initially suspected of having HD [10, 11].

Additionally, obtaining a reliable family history can be challenging, as relevant information is often not readily apparent. On closer inquiry, previously unrecognized factors such as early parental death, unclear ancestry, or psychiatric illness may be suggestive of a hereditary background. These can possibly provide indirect clues of a hereditary burden as HD patients show a high suicide rate related to the psychiatric comorbidities, which can often precede the first motor symptoms [12]. Consequently, the diagnostic utility of family history is uncertain and may even be misleading in some cases [13]. To address this, we performed a retrospective analysis to evaluate the spectrum and frequency of hereditary choreatic disorders lacking a positive family history for HD or chorea-related syndromes.

## Methods

We conducted a retrospective analysis of clinical records from 81 patients who presented with chorea as a prominent clinical feature at the Movement Disorders Outpatient Clinic, Medical University of Vienna. Patients were included between 2013 and 2024, coinciding with the implementation of the current electronic health record system. Clinical data including motor phenomenology, family history, and medication history were systematically collected. All patients underwent comprehensive laboratory testing to exclude secondary causes, including complete blood count with blood smear (for acanthocytes), serum electrolytes, liver, kidney, and thyroid function tests, serum, and urine copper, ceruloplasmin, autoimmune antibodies, and vasculitis markers. Neuroimaging with brain MRI was performed in all patients, supplemented by lumbar puncture, nuclear medicine studies, or CT scans of the thorax, abdomen, or pelvis as clinically indicated. After excluding secondary causes, genetic testing was performed using a targeted gene panel including *HTT*, *SCA1*, *SCA2*, *SCA3*, *SCA6*, *SCA17*, *C9ORF72*, *DRPLA*, and *HDL2*. In cases with negative panel results, further genetic evaluation was conducted using whole-exome (WES) or whole-genome sequencing (WGS). Variants from these genetic tests were classified as (i) pathogenic (P), (ii) likely pathogenic (LP) and variants of unknown significance (VUS) according to the ACMG criteria[14]. The study was approved by the Ethics Committee of the Medical University of Vienna (EK Nr: 1644/2025) and was conducted in accordance with the principles of the Declaration of Helsinki.

### Data availability

Anonymized data not published within this article will be made available by request from any qualified investigator.

### Statistics

A descriptive data analysis was carried out using the IBM-SPSS.23 program. The collected parameters are shown as percentages or absolute figures in Table 1.

**Table 1:** Presentation of main features of patients with negative family history

Overview of patients with negative family history	
Number of patients (n)	n = 44
Female (n, %)	n = 25 (56,8%)
Age of onset (y)	53 years (1–82)
Movement disorders (n, %)	Generalized chorea: n = 40 (90,91%) Hemichorea: n = 4 (9,09%) Orolingual Dyskinesia: n = 9 (20,45%) Dystonia: n = 3 (6,82%) Tremor: n = 3 (6,82%) Ataxia: n = 5 (11,36%) Parkinsonism: n = 2 (4,55%) Myoclonus: n = 1 (2,27%)
Aetiology (n, %)	hereditary: n = 30 (68,18%) HD: n = 20 (66,67%) C9orf72: n = 1 (3,33%) SCA17: n = 2; (6,67%) WD: n = 2 (6,67%) Other genetic causes: n = 5 (16,67%) acquired: n = 8 (18,18%) autoimmune: n = 3 (37,5%) paraneoplastic: n = 0 (0%) metabolic: n = 1 (12,5%) infectious: n = 0 (0%) drug-induced: n = 2 (25%) hypoxic: n = 1 (12,5%) Fahr’s Disease: n = 1 (12,5%) unknown: n = 6 (13,64%)

*n* numbers; *y*-years; *HD* Huntington’s Disease; *C9orf72* C9orf72-related neurodegeneration; *SCA17* Spinocerebellar Ataxia Type 17; *WD* Wilson’s Disease

## Results

**Fig. 1:**
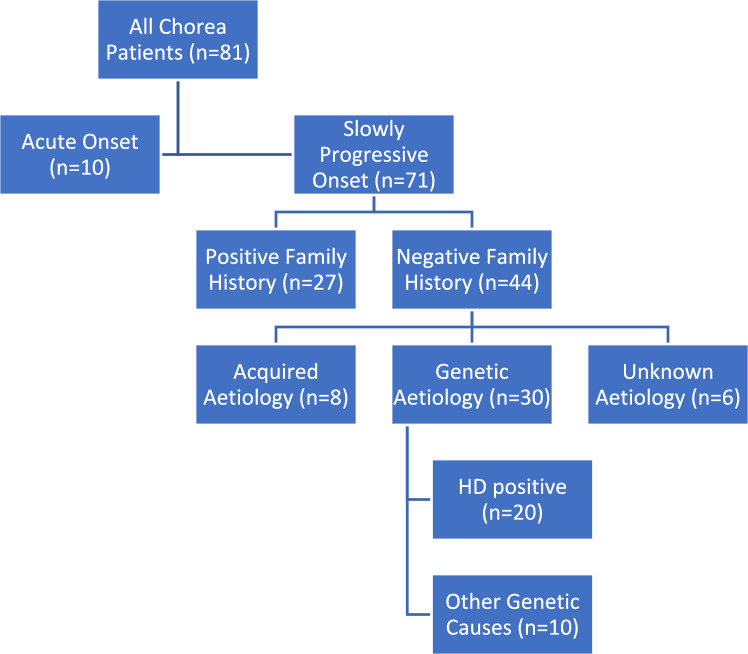
Representation of the entire chorea cohort as a flowchart

A total of 81 patients with chorea consulted the movement disorder outpatient clinic of the Medical University of Vienna between 2013 and 2024 (Fig. 1). Among them, 10 patients presented with sudden clinical onset associated with a new brain lesion. The remaining 71 patients had a slowly progressive course. In 27 patients, there was a clear positive family history of HD (n = 25) or a chorea-related syndrome (WDR73 associated Galloway-Mowat Syndrome n = 1, NKX2-1 associated benign chorea n = 1), which was also confirmed in those individuals. In the subgroup of 44 patients presenting with a negative family history and slowly progressive chorea, laboratory and imaging studies identified secondary etiologies in 8 cases: autoimmune (n = 2 antiphospholipid antibody syndrome; n = 1 paraproteinemia; in total n = 3), metabolic (hyperthyroidism; n = 1), drug-induced (n = 2), Fahr’s Disease (n = 1) and hypoxic (n = 1) (see Table 1).

Among the remaining 36 patients, genetic testing yielded a positive result in 30 cases. The most common genetic etiology was Huntington’s Disease (HD, n = 20), followed by Spinocerebellar Ataxia Type 17 (SCA17, n = 2), Wilson’s Disease (WD, n = 2), Ataxia with Oculomotor Apraxia Type 2 (AOA2, n = 1), C9orf72-related Neurodegeneration (n = 1), Choreoacanthocytosis (ChAc, n = 1), KMT2B-related Dystonia (n = 1), ERCC4-related Neurodegeneration (n = 1), and Glutaric Aciduria Type 1 (GA1, n = 1).

In six patients, the underlying etiology still remains undetermined. Although WES identified in one patient a heterozygous TMEM151A variant (NM_153266.4: c.10G > A, p.Asp4Asn), a gene associated with autosomal dominant paroxysmal kinesigenic dyskinesia type 3, this variant was considered unlikely to be causative due to the discordance between the patient’s phenotype—characterized almost exclusively by orofacial dyskinesia without involvement of other body regions—and the known clinical presentation of the disorder, as well as its uncertain clinical significance (ACMG class 3, VUS). Interestingly, four patients initially presented with a Hemichorea, in three of them the disease later evolved into a generalized chorea with persistent unilateral predominance. All patients underwent at least one cerebral MRI, FDG-PET imaging, and cerebrospinal fluid analysis. An abnormal FDG-PET finding was observed in only one patient, demonstrating bilateral hypometabolism involving the basal ganglia, thalamus, and insular cortex. The patient died shortly thereafter, so further diagnostic investigations beyond standard genetic panel testing were not possible. In two patients, the initial genetic workup had been completed more than five years ago. Given the substantial advances in genomic technologies since then, repeat WES or additional WGS may be justified.

In assessing family history, particular attention was given to reports of early deaths and psychiatric disorders among relatives. An early death (defined as occurring before age 50) of a first degree relative was documented in 10 of the 20 patients diagnosed with Huntington’s disease. Reported causes of death included accidents (e.g., avalanche, staircase fall) and medical conditions such as poliomyelitis, myocardial infarction, and stroke. Notably, no suicides in the family were reported. The reported causes of death were not considered typical indications of a premotor syndrome of possible HD and were therefore classified as unremarkable family history. In two cases, a parental history of affective disorder was identified, which may represent an indirect indicator of psychiatric manifestations associated with HD. It is also important to note that there are no intra-familial relationships among the patients.

### Huntington’s disease

All 20 patients showed a generalized choreatic movement disorder with a gradual onset and slowly progressive course. Beyond that, no other `red flags´ could be identified that would have led us to think of another form of genetic chorea [8]. The mean age of onset was 52 years (52 ± 12 years) and there was a female predominance of 70%. Compared to the HD patients with a positive family history these patients had on average a lower trinucleotide repeat expansion number (43 CAG repeats vs. 45 CAG repeats, p = 0.037, one-sided t-test, uncorrected) and a correspondingly later disease onset (52 years vs. 42 years, p = 0.014, one-sided t-test, uncorrected), which was consistent with previous studies [15].

### Other genetic chorea

In comparison with the previously mentioned HD patients, almost all patients with another underlying genetic mutation showed further clinical signs. Most frequently, ataxia, gait disorders, various forms of tremor or a primarily facio-bucco-linguo chorea were found.

#### Spinocerebellar ataxia type 17 (SCA17)

Two non-related female patients were diagnosed with SCA17, both presenting with disease onset in the sixth or seventh decade of life (age of onset [AOO] 65 and 74 years). In addition to mild generalized chorea, one patient exhibited pronounced bilateral resting, postural, and action tremor with gait impairment, while the other demonstrated limb ataxia and saccadic eye movement. Neither patient developed psychiatric symptoms or dementia, which are typically associated with this SCA subtype [16]. Both patients exhibited CAG repeat lengths ranging from 41 to 48 repeats, a range known to be associated with incomplete penetrance. Recent studies have suggested that an accompanying heterozygous co-mutation in the *STUB1* gene may contribute to clinical manifestation in such cases, especially those with a HD-like phenotype [17, 18]. However, analysis for co-occurring *STUB1* variants was not performed in our patients, as the potential modifying role of *STUB1* mutations in SCA17 had not yet been described at that time. In the future, assessment of *STUB1* variants may contribute to more accurate molecular diagnosis and genetic counseling in patients with SCA17.

#### Wilson’s disease (WD)

Two genetically confirmed cases of WD showed heterogeneous clinical phenotypes. A male patient (AOO 13 years) presented with generalized choreo-dystonic hyperkinesia, gait disturbance, rigidity, and tremor (*ATP7B*: c.2299insC/p.G1341D). In contrast, a female patient (AOO 62 years) had an atypical presentation, with longstanding depression since childhood and later-onset orofacial dyskinesia and distal chorea. Genetic testing identified a pathogenic missense mutation (*ATP7B*: c.3140A > T, p.D1047V) [19]. Typical hepatic signs and Kayser–Fleischer rings were absent, consistent with the described presentation of isolated neurological manifestations in late-onset WD [20].

#### Ataxia with oculomotor apraxia type 2 (AOA2)

One patient (male, AOO 24 years) had generalized chorea and a pronounced sensorimotor polyneuropathy with stance and gait ataxia. In this patient, two rare mutations in the *SETX* gene (*SETX*: c.5825 T > C, p.lle1942Thr and an essential splice site mutation, hg38: 9–132,295,871-C-A, NM_015046.7 c.6106 + 1G > T, p.?) (Exon15/Intron15) were detected and confirmed as pathological in a further RNA-analysis. Consequently, AOA2 was diagnosed [21]. The patient did not exhibit the typical feature of oculomotor apraxia, which is reported to be absent in approximately 50% of cases [22].

#### C9orf72-related neurodegeneration

A female patient (AOO 72 years) exhibited isolated generalized chorea and mild bradykinesia. Genetic testing revealed a *C9orf72* hexanucleotide expansion. Although typically associated with frontotemporal dementia (FTD) or amyotrophic lateral sclerosis (ALS), *C9orf72* mutations can also manifest with various movement disorders including chorea [23].

#### Choreoacanthocytosis (ChAc)

A female patient (AOO 64 years) had an isolated chorea in her left lower limb and orofacial dyskinesia (lip smacking, licking). Based on the presence of two heterozygous *VPS13A* variants of uncertain significance (missense mutation NM001018038.2:c.9128A > G and synonymous mutation NM_001018038.2:c.7533A > G) and acanthocytosis on peripheral smear A ChAc was suspected. However, additional typical features of ChAc (e.g., caudate atrophy, hyperCKemia, epilepsy, or peripheral neuropathy) were absent [24]. Therefore, the diagnosis of ChAc remains preliminary at present, as no functional studies confirming the pathogenicity of the two identified VUS are currently available.

#### KMT2B-related dystonia (dystonia type 28)

A male patient (AOO 13 years) developed progressive generalized choreo-dystonia, dysarthria, and later myoclonus, with difficulties in chewing and swallowing. A pathogenic frameshift mutation in *KMT2B* (c.5697del, p.Thr1900Glnfs*34) was identified [25]. The patient received a deep brain stimulation (DBS) at the age of 21, which led to a significant and so far, persisting improvement of the hyperkinesia [26].

#### ERCC4-related neurodegeneration (xeroderma pigmentosum group F)

A male patient (AOO 45 years) initially presented with cognitive decline and was evaluated for dementia. Over time, chorea, gait ataxia, and dysarthria developed. Next-generation sequencing revealed a likely pathogenic variant (missense mutation NM_005236.3:c.2395C > T) and a pathogenic variant (nonsense mutation NM_005236.3:c.202G > T)) in the *ERCC4* gene. Retrospective history-taking uncovered lifelong photosensitivity, supporting the diagnosis of Xeroderma Pigmentosum group F [27, 28].

#### Glutaric acidemia type 1 (GA1)

A female patient (AOO 9 years) was diagnosed with GA1 after developing generalized trunk-dominant choreoathetosis and mild orofacial dyskinesia. The diagnosis had previously been missed at birth. Notably, the patient did not develop dystonia or epilepsy, which are common neurological features of GA1 [9, 29].

## Discussion

This retrospective analysis of data from a single-center movement disorders clinic in Vienna highlights the broad and heterogeneous etiological spectrum of chorea, emphasizing the diagnostic complexity associated with these disorders. The aim was to assess the role and diagnostic yield of genetic testing specifically in patients presenting with choreatic movement disorders and a negative family history. This focus was particularly relevant, as we found no studies explicitly evaluating the prevalence of genetically confirmed chorea in this patient subgroup. Existing literature has primarily concentrated either on Huntington’s disease cohorts, non-hereditary chorea, or has not systematically accounted for the role of family history in the diagnostic process [11, 15, 30–34].

The most striking finding of this study is the high diagnostic yield of genetic testing in patients with slowly progressive chorea despite a negative family history, even when indirect indicators such as suicides among relatives are considered. After exclusion of secondary causes, a monogenic etiology was identified in 30 of these 36 patients (68.18%). The majority were diagnosed with HD (n = 20, 64.52%), while other genetic causes such as Wilson’s disease, Chorea-Acanthocytosis, SCA17, and C9orf72-expansion disorders were each detected in individual cases. Given the limited number of patients in each diagnostic category, subgroup analyses were not feasible due to insufficient statistical power. Notably, about 44% (n = 20) of our overall HD patients (n = 45) had a negative family history—significantly higher than the 6–24% reported in previous studies [15, 30, 31]. In our cohort, the evaluation of family history revealed no reported cases of suicide, and affective disorders were the only psychiatric comorbidity identified among relatives in two patients. These findings underline the importance of comprehensive genetic testing even in apparently sporadic cases and suggest a higher-than-expected prevalence of monogenic chorea in this population [32, 34].

However, several limitations must be considered. As our movement disorders outpatient clinic provides care for a broad range of conditions, it has a particular emphasis on advanced therapies for Parkinson’s Disease and Essential Tremor, rather than on disorders such as chorea. As a result, our overall cohort of chorea patients comprised 81 patients over a 12-year period (2013–2024), representing a small proportion of the total 2,705 outpatient visits during that time. Additionally, it is important to note that patients with acute-onset chorea—such as those related to hyperglycemia, stroke, or intracerebral hemorrhage—were typically managed in inpatient settings due to the transient and self-limiting nature of their symptoms and thus were not captured in our outpatient dataset. Consequently, our cohort predominantly includes patients with persistent choreatic movement disorders, often requiring symptomatic pharmacological treatment and long-term neurological follow-up. In addition, as a tertiary referral center, our cohort may be enriched for complex or atypical cases, potentially inflating the observed diagnostic yield. Therefore, our findings may not be directly generalizable to all clinical settings.

From a diagnostic perspective, our findings support a low threshold for genetic testing in patients with unexplained, slowly progressive chorea, even in the absence of a family history. This is important to emphasize, because several factors can obscure a positive family history: especially early deaths, intermediate alleles, alleles with reduced penetrance, late-onset diseases, non-paternity and adoptions can be misleading. Instead, genetic testing should be considered once secondary causes have been systematically excluded, particularly in the presence of additional neurological or psychiatric features. Given the high diagnostic yield observed, early genetic evaluation may reduce diagnostic delay, prevent unnecessary investigations, and facilitate appropriate counseling, prognostic stratification, and access to disease-specific management or clinical trials.

While the current stepwise diagnostic pathway—beginning with targeted testing for Huntington’s disease repeat expansions, followed by gene panels and subsequently exome sequencing—remains a cost-effective and clinically established approach, evolving sequencing technologies are increasingly reshaping this algorithm [11, 13]. In particular, whole-genome sequencing is emerging as a promising, future-oriented alternative due to its ability to detect single nucleotide variants, copy number variants, structural variants, and repeat expansions within a single assay. In routine clinical practice, short-read sequencing technologies remain the most widely used approach; however, their limited read length constrains the reliable detection of a substantial proportion of structural variation and results in inaccessibility of large genomic regions, particularly those that are repetitive, GC-rich, or structurally complex. In this context, emerging long-read sequencing technologies represent a promising alternative, enabling a more comprehensive characterization of complex genomic architecture and previously inaccessible regions of the genome [35]. Their integration into clinical workflows therefore has the potential not only to increase diagnostic yield in currently unresolved cases of chorea, but also to meaningfully reshape or ultimately replace existing stepwise diagnostic algorithms as technical accessibility and clinical implementation continue to advance.

## Conclusion

The differential diagnosis of chorea remains a major challenge due to the wide variety of causes. While acute or subacute forms can often be assigned more quickly to vascular, metabolic, or infectious causes, slowly progressive forms are more complex and frequently require extensive evaluation. Genetic choreatic syndromes can present with diverse and overlapping clinical features, and their phenotypic expression may vary significantly depending on age of onset. This retrospective analysis challenges the traditional assumption that a negative family history substantially lowers the pre-test probability of a genetic diagnosis. Therefore, reliance on family history alone would have resulted in missed or delayed diagnosis in a substantial proportion of patients.

Owing to scientific advances, particularly in genetic diseases, an increasing number of mutations have been linked to choreatic movement disorders. This in combination with a wider availability and accessibility of genetic tests helped to solve a great number a multitude of patient cases with chorea. Our cohort illustrates that monogenic chorea can occur even in the absence of a documented family history. Therefore, a negative family history should not be considered an exclusion criterion for genetic evaluation. As new genetic variants continue to be discovered, previously unsolved cases may eventually be diagnosed. These findings also support the growing view that the true prevalence of Huntington’s disease and related genetic chorea is likely underestimated [31].

## Data Availability

The principal author Tekla Anna Fodor has full access to the data used in the analyses in the manuscript and takes full responsibility for the data, the analyses and interpretation, and the conduct of the research.
